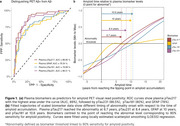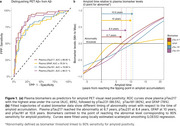# Mapping Plasma Biomarker Progression Against the Amyloid Clock

**DOI:** 10.1002/alz70856_106593

**Published:** 2026-01-07

**Authors:** Marina Bluma, Konstantinos Chiotis, Marco Bucci, Irina Savitcheva, Ilaria Pola, Wiebke Traichel, Kübra Tan, Guglielmo Di Molfetta, Anna Matton, Miia Kivipelto, Andrea L. Benedet, Nicholas Ashton, Kaj Blennow, Henrik Zetterberg, Agneta K Nordberg

**Affiliations:** ^1^ Department of Neurobiology, Care Sciences and Society, Center for Alzheimer Research, Division of Clinical Geriatrics, Karolinska Institutet, Stockholm, Sweden; ^2^ Department of Neurobiology, Care Sciences and Society, Division of Clinical Geriatrics, Center for Alzheimer Research, Karolinska Institutet, Stockholm, Sweden; ^3^ Medical Radiation Physics and Nuclear Medicine, Section for Nuclear Medicine, Karolinska University Hospital, Stockholm, Sweden; ^4^ Department of Psychiatry and Neurochemistry, Institute of Neuroscience and Physiology, The Sahlgrenska Academy, University of Gothenburg, Mölndal, Sweden; ^5^ Institute of Neuroscienace and Physiology, University of Gothenburg, Mölndal, Västra Götaland, Sweden; ^6^ Department of Neurobiology, Care Sciences and Society, Centre for Alzheimer Research, Division of Clinical Geriatrics, Karolinska Institutet, Stockholm, Sweden; ^7^ Theme Inflammation and Aging, Karolinska University Hospital, Stockholm, Sweden; ^8^ Department of Psychiatry and Neurochemistry, Institute of Neuroscience and Physiology, University of Gothenburg, Mölndal, Sweden

## Abstract

**Background:**

Plasma biomarkers are potential candidates for screening patients for anti‐amyloid therapies. Although early changes have been reported, the duration of Aβ‐accumulation afetr which these changes become abnormal enough to identify Aβ+ individuals has yet to be determined.

**Method:**

Data were acquired from a cohort assessed at the Memory Clinic, Karolinska University Hospital, Stockholm, Sweden (*N* = 132). Plasma biomarkers (pTau‐isoforms, GFAP) were analysed with NULISAseq™ CNS. Buildong up on the parameters of a model of Aβ‐accumulation estimated by Schindler et al., 2021, we calculated the time required (AmyloidTime) to reach a specific brain amyloid load on PET. We then estimated AmyloidTime linked with a threshold of biomarker becoming significantly abnormal to detect Aβ‐positivity with 90% sensitivity. By subtracting AmyloidTime from each patient's age, we estimated their Aβ‐accumulation onset ('age‐of‐Aβ‐onset'), identifying young accumulators as those with age‐of‐Aβ‐onset under 50 years, which would correspond to a minimum of 15 years of Aβ‐accumulation at age 65.

**Result:**

The AmyloidTime corresponding to 90% sensitivity for detecting Aβ+ with the visual read was 2 years prior to the tipping point in Aβ‐accumulation. The AmyloidTime required for plasma biomarkers become abnormal enough to provide 90% sensitivity in identifying Aβ+ PET scans were: 6.7 years for pTau217, 8.4 – for pTau231,10 ‐ for GFAP, 10.6 ‐ for pTau181 (Figure 1). Plasma pTau217, pTau231, pTau181 levels, but not GFAP, were significantly higher in young Aβ‐accumulators, even after adjusting for the effect of Aβ‐load or ‐Time (Figure 2).

**Conclusion:**

Plasma pTau217 enables 90% sensitivity in identifying Aβ+ individuals at 6.7 years after reaching the point of accelerated Aβ‐accumulation. Younger Aβ‐accumulators showed higher levels of pTau isoforms in plasma, suggesting an exacerbation of tau pathology in these individuals, which is consistent with a more aggressive pathology in this age group.